# Angiocholite sur kyste hydatique hépatique fistulisé dans la voie biliaire principale: à propos de 2 cas

**DOI:** 10.11604/pamj.2022.42.62.30130

**Published:** 2022-05-23

**Authors:** Hanane Delsa, Najwa Benslima, Imane Rahmouni, Yasmine Cherouaqi, Amine Benfaida, Leila Abdallaoui Maane, Mohamed Reda Cherkaoui Jaouad, Fatima Belabbes, Anass Nadi, Fedoua Rouibaa

**Affiliations:** 1 Service de Gastroentérologie, Hôpital Cheikh Khalifa, Casablanca, Maroc,; 2 Faculté de Médecine, Université Mohammed VI des Sciences de la Santé (UM6SS), Casablanca, Maroc,; 3 Service de Radiologie, Hôpital Cheikh Khalifa, Casablanca, Maroc

**Keywords:** Kyste hydatique, angiocholite, sphinctérotomie, cas clinique, Hydatid cyst, angiocholitis, sphincterotomy, case report

## Abstract

Le kyste hydatique est une zoonose qui touche fréquemment le foie, qui sévit à l´état endémique dans plusieurs pays comme le Maroc. L´hydatidose hépatique peut se compliquer d´une angiocholite dont le traitement de choix est actuellement la cholangio-pancréatographie rétrograde endoscopique avec sphinctérotomie. Nous rapportons deux cas cliniques d´angiocholite sur kyste hydatique hépatique fistulisé dans la voie biliaire principale qui ont été traités par voie endoscopique avec une évolution favorable. Le diagnostic précoce et la prise en charge adéquate permettent l´amélioration du pronostic de ces patients.

## Abstract (en)

Hydatid cyst is a zoonosis that frequently affects the liver, which is endemic in several countries such as Morocco. The hepatic hydatidosis can be complicated by angiocholitis, currently, the treatment of choice is endoscopic retrograde cholangio-pancreatography with sphincterotomy. We report two clinical cases of angiocholitis on hepatic hydatid cyst fistulised in the main bile duct which were treated endoscopically with a favourable outcome. Early diagnosis and adequate management can improve the prognosis of these patients.

## Introduction

L´hydatidose ou échinococcose hydatique ou le kyste hydatique (KH) est une anthropozoonose non immunisante provoquée par l´ingestion accidentelle d´œufs d´*Echinococcus granulosus* provenant du chien, hôte définitif habituel du ténia échinocoque. Cette maladie parasitaire bénigne peut toucher tous les organes mais plus fréquemment le foie. Le KH est endémique dans de nombreux pays du pourtour méditerranéen y compris le Maroc et constitue un véritable problème de santé publique. Sa prévalence peut atteindre 5% à 10% dans certaines régions [1] avec un vrai impact économique surtout dans les pays en voie de développement comme le Maroc [2]. La complication la plus redoutable du kyste hydatique du foie (KHF) est la rupture dans les voies biliaires qui peut engendrer une angiocholite et un état de septicémie sévère engageant le pronostic vital. Le KHF fistulisé a connu plusieurs avancées thérapeutiques d´une chirurgie lourde vers un drainage endoscopique permettant une moindre morbi-mortalité. Nous rapportons deux cas cliniques admis pour angiocholite sur rupture de kyste hydatique hépatique drainée par une cholangio-pancréatographie rétrograde endoscopique (CPRE) avec sphinctérotomie.

## Patient et observation

### Observation N°1

**Information du patient:** malade âgé de 57 ans, ayant comme antécédent une appendicectomie il y a 1 an, opéré à 2 reprises pour kyste hydatique du foie il y a 21 ans. Admis aux urgences pour des douleurs abdominales avec vomissements post-prandiaux, aggravés par l´apparition d´un subictère et des urines foncées, le tout évoluant dans un contexte de fièvre chiffrée à 39°C avec des frissons.

**Résultats cliniques:** l´examen retrouve un patient conscient, subictérique et stable sur le plan hémodynamique et respiratoire. L´examen abdominal est sans anomalies.

**Démarche diagnostique:** bilan biologique objective une hyperleucocytose à 15000/mm à prédominance polynucléaires neutrophiles à 13200/mm, une cytolyse et cholestase à bilirubine conjuguée, une protéine C réactive à 110 avec une procalcitonine élevée.

**Figure 1:**
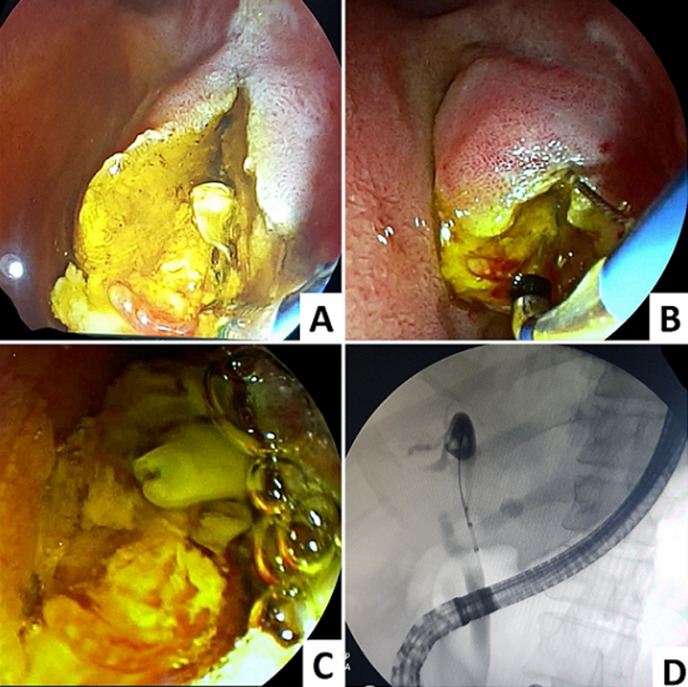
drainage endoscopique du KH rompu dans la voie biliaire principale avec issue de kystes filles; (A et B) sphinctérotomie élargie, C) vue endoscopique du matériel hydatique; D) vue scopique du ramonage de la voie biliaire principale à l´aide d´un ballon d´extraction

**Intervention thérapeutique:** une échographie abdominale a montré un nodule hépatique du segment 1 évoquant un KH stade IV avec dilatation de la voie biliaire principale (VBP). La cholangiographie par résonance magnétique (BILI-IRM) a confirmé la présence de matériel évoquant un kyste hydatique rompu dans les voies biliaires. Devant ce tableau septicémique, une antibiothérapie a été démarrée dès son admission. Une CPRE a été réalisée avec opacification de la voie biliaire principale qui est dilatée avec présence d´un matériel endoluminal. Après une sphinctérotomie élargie (Figure 1A et B), le ramonage de la voie biliaire principale à l´aide d´un ballon d´extraction (Figure 1C) a permis l´issu du matériel hydatique (Figure 1D).

**Suivi:** l´évolution était favorable avec disparition de la septicémie et des symptômes ainsi que la normalisation du bilan hépatique. Le contrôle échographique a montré la persistance d´une petite cavité résiduelle, un traitement à base d´Albendazole a été instauré avec bonne évolution.

### Observation N°2

**Information du patient:** malade âgée de 54 ans, connue hypertendue, opérée pour kyste hydatique du foie il y a 22 ans, consulte pour une douleur de l´hypochondre droit avec un ictère d´allure cholestatique. Le tout évoluant dans un contexte de frissons avec sensation fébrile non chiffrée.

**Résultats cliniques:** l´examen retrouve une patiente consciente, stable sur le plan hémodynamique, ictérique avec une sensibilité de l´hypochondre droit. Le reste de l´examen est sans particularités.

**Figure 2:**
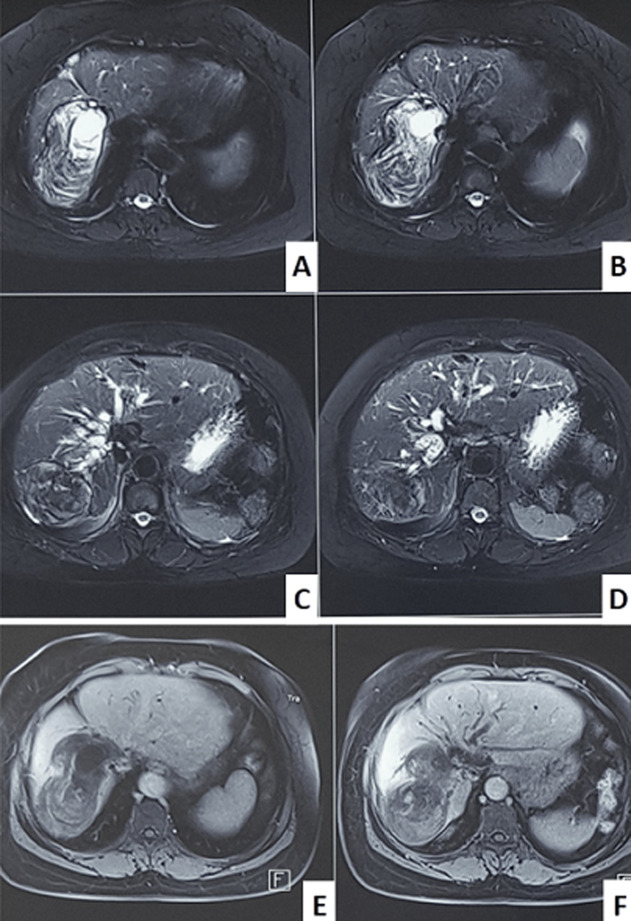
coupe axiale de la Bili-IRM en séquences pondérées en T2 avec saturation du signal de la graisse (A, B, C et D), montrant une masse solido-kystique au segment VII associée à une dilatation des voies biliaires intra-hépatiques, (E et F) absence de rehaussement après injection de produit de contraste

**Figure 3:**
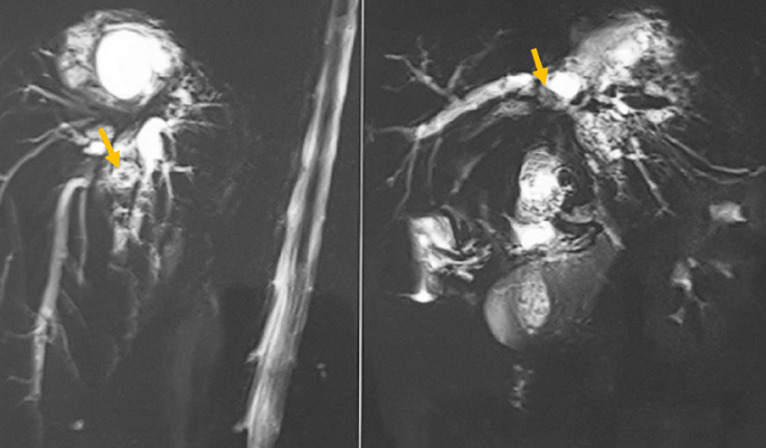
reconstruction 3D d´une Bili-IRM montrant une dilatation des voies biliaires intra-hépatiques qui renferment un matériel endoluminal (flèche jaune)

**Démarche diagnostique:** le bilan biologique objective une cytolyse avec une cholestase associée à une hyperbilirubinémie au dépend de la bilirubine conjuguée, la protéine C réactive était également élevée. La Bili-IRM a confirmé la récidive du kyste hydatique classé II siégeant au niveau du foie droit mesurant à 10,2 x 5,6cm avec des signes de rupture dans la VBP qui est dilatée à 22mm (Figure 2, Figure 3).

**Figure 4:**
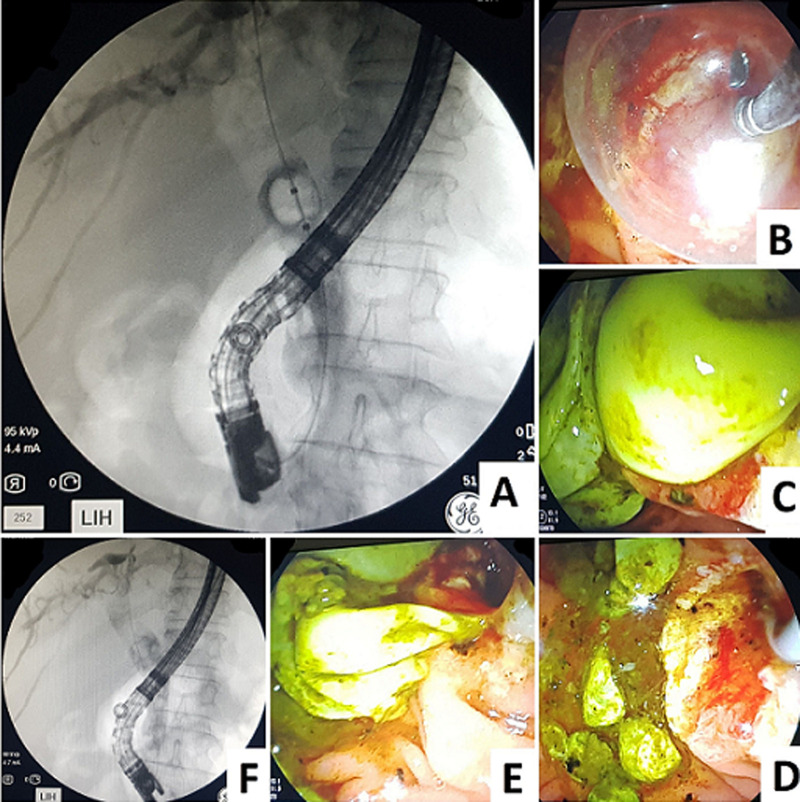
image de CPRE permettant le drainage endoscopique du KH rompu dans la voie biliaire principale; A) vue scopique du ballon d´extraction; B) vue endoscopique du ballon d´extraction; C, D et E) issue de plusieurs membranes hydatiques; F) la vacuité de la voie biliaire principale en scopie en fin de procédure

**Intervention thérapeutique:** un traitement endoscopique a été proposé avec une sphinctérotomie et drainage endoscopique par ballon d´extraction (Figure 4A, B) avec issue de plusieurs membranes hydatiques (Figure 4 C, D et E), sous couverture antibiotique. Le contrôle scopique a confirmé la vacuité de la VBP (Figure 4F).

**Suivi:** cette thérapeutique a permis la disparition des signes cliniques ainsi que la normalisation du bilan hépatique.

**Perspective des patients:** pendant leurs hospitalisations, les patients étaient satisfaits des soins reçus. Après le traitement, ils étaient optimistes concernant l´évolution de leurs états de santé.

**Consentements des patients:** le consentement éclairé a été obtenu des malades concernant l´utilisation des images.

## Discussion

L´échinococcose kystique existe sur tous les continents sauf l´Antarctique. Dans les régions endémiques, leurs taux d´incidence chez l´homme peuvent dépasser 50 pour 100 000 personnes-années, et la prévalence peut atteindre 5% à 10% dans certaines régions [1]. En 2015, le groupe de référence de l´Organisation Mondiale de la Santé sur l´épidémiologie des maladies d´origine alimentaire a estimé que l´échinococcose était à l´origine de 19,300 décès [1]. Par conséquence l´hydatidose a un impact économique considérable surtout dans les pays en voie de développement comme le Maroc [2]. En 2007, le Maroc a instauré un programme national de lutte contre l´hydatidose (PNLH), dont le but est de réduire l´incidence de cette maladie et la contrôler à l´échelle national [3]. Le Maroc a aussi entrepris un projet de décentralisation des techniques diagnostiques et thérapeutiques et de promotion de la stratégie Ponction-Aspiration-Injection-Réaspiration (PAIR) dans les zones rurales et d´hyper-endémie afin de mieux traiter cette maladie et surtout prévenir les complications.

Les complications du kyste hydatique du foie peuvent être inaugurales et variables selon les régions et les séries. Elles sont largement dominées par les ruptures kystiques dans les voies biliaires, qui constitue une complication assez grave et fréquente (17 à 44%) des kystes hydatiques opérés [4]. Cette rupture peut engendrer des tableaux polymorphes d´angiocholite aiguë et/ou de suppuration profonde. Dans une série marocaine incluant 536 malades opérés pour KHF, 22,38% des cas se sont compliqués d´une rupture dans les voies biliaires manifestée par une angiocholite dans 39,16% des cas [5]. Elle survient souvent quelques années après la chirurgie du kyste hydatique, néanmoins quelques cas ont été rapportés après une dizaine d´année [6], nos patients ont présenté cette complication après 20 ans de la résection chirurgicale du KH, une situation assez inhabituelle. Le diagnostic repose sur l´imagerie médicale, L´échographie et la tomodensitométrie permettant de préciser les caractéristiques du kyste hydatique hépatique ainsi que visualiser les débris hydatiques dans la VBP. Les classifications les plus utilisées sont celle de Gharbi [7] et celle de l´Organisation Mondiale de la Santé [8]. Néanmoins, la bili-IRM constitue la technique de choix pour l´exploration des voies biliaires, nos malades ont pu bénéficier de cet examen qui a permis d´affirmer le diagnostic d´une rupture du KHF dans la VBP.

Pendant longtemps, le traitement le plus proposé était la chirurgie, avec une polarisation parfois dogmatique du débat technique entre approches dites conservatrices (résection du dôme saillant) et radicales (péri-kystectomies voire hépatectomies) [9]. Au milieu des années 80, une nouvelle technique percutanée a été développée qui est la PAIR sous guidage échographique [10]. Cette technique est une méthode moins invasive, moins traumatisante, et surtout moins coûteuse que la chirurgie classique. Elle doit être proposée chez les patients inopérables ou refusant la chirurgie. Depuis quelques années, la CPRE avec sphinctérotomie endoscopique est une méthode sure et efficace qui est devenue la méthode de choix pour le traitement des KHF rompus avec fistule bilio-bronchique ou bilio-hépatique [11-14]. L´endoscopie interventionnelle joue aussi un rôle majeur dans la gestion des urgences et des complications biliaires avec une morbi-mortalité moindre que la chirurgie [9]. Ce traitement endoscopique a également montré son efficacité en préopératoire pour la prise en charge des kystes hydatiques rompus avec fistule bilio-bronchique. En combinant l´amélioration des mesures de préparation préopératoire et la correction des troubles biologiques, une thoracotomie exclusive est désormais possible avec des résultats satisfaisants permettant une cicatrisation complète de la fistule dans certains cas [11]. Le traitement endoscopique a été effectué chez nos deux patients, ce qui a permis la gestion de l´angiocholite en urgence avec des résultats satisfaisants et sans incidents post-procédures.

En pratique, les données cliniques permettent d´affiner les indications et les modalités de prise en charge. Une stratégie thérapeutique multimodal concertée doit être instaurée qui va inclure des traitements percutanés, médicaux et endoscopiques [9].

## Conclusion

La CPRE avec sphinctérotomie endoscopique est actuellement une procédure utile et sûre pour le diagnostic mais surtout le traitement des complications biliaires de l´hydatidose hépatique. Elle permet également la gestion en urgence des angiocholites qui est la principale complication retrouvée chez nos patients. L´efficacité du traitement endoscopique est confirmée avec une morbi-mortalité moindre que la chirurgie radicale, par conséquence il doit être proposé en première intention et constitué le traitement de choix des angiocholites sur kyste hydatique rompu dans la voie biliaire principale.

## Remerciements

Nous remercions tout le personnel d´endoscopie digestive de l´Hôpital Universitaire Cheikh Khalifa Casablanca, Maroc.
